# A Digitally Capable Aged Care Workforce: Demands and Directions for Workforce Education and Development

**DOI:** 10.2196/54143

**Published:** 2025-04-02

**Authors:** Kathleen Gray, Kerryn Butler-Henderson, Karen Day

**Affiliations:** 1 Centre for Digital Transformation of Health University of Melbourne Melbourne Australia; 2 School of Nursing, Paramedicine, and Healthcare Sciences Charles Sturt University Bathurst Australia; 3 Health Systems Group University of Auckland Auckland New Zealand

**Keywords:** aged care, digital health, digital literacy, education, older adults, professional development, digital transformation, digital resources, users, community, learning, support, safe, ethical, satisfaction

## Abstract

As the aged care sector undergoes digital transformation, greater attention is needed to development of digital health capability in its workforce. There are many gaps in our understanding of the current and future impacts of technology on those who perform paid and unpaid aged care work. Research is needed to understand how to make optimal use of both digital resources and human resources for better aged care. In this Viewpoint, we reflect on a workshop held during an international conference that identified shared concepts and concerns to shape further research into workforce capability. Digital technologies and digital data can increase quality of care in a system that operates through partnerships among service providers, service users, and community members. To realize this potential, digital health learning and development are needed in the aged care workforce. As digital dimensions of aged care services expand, the sector needs clearer direction to implement approaches to workforce learning and development. These must be appropriate to support the safe and ethical performance of care work and to increase the satisfaction of those who care and those for whom they care.

## Why Focus on a Digitally Capable Aged Care Workforce?

The aged care sector is in the spotlight around the world due to the increasing health and well-being needs of aging populations. Examples are the Australian Royal Commission into Aged Care Quality and Safety [1], Japan’s Social Welfare for the Elderly Act [2], and Germany’s national report examining the human rights of older persons in long-term care [3]. Comparisons of aged care systems internationally focus on a variety of issues [4,5].

In such reports, the aged care workforce emerges as an essential factor. One part of this workforce is paid, structured, and regulated (eg, registered nurses) and formally counted in the health and care workforce. Another part of this workforce is informal—often unpaid and not structured or regulated (eg, relatives as unpaid caregivers) but contributing in incalculable ways [6]. Both parts operate under conditions that can be inequitable, inefficient, uncertain, and unsatisfactory. The overall situation of the aged care workforce demands widespread improvements in recruitment and retention, practice standards, skill levels, recognition, and reward [7].

Digital maturity in aged care service provider organizations lags behind mainstream health care services; there are differences between the two also in terms of information needs, technologies of interest, and administration. The medico-legal record for aged care services works differently from hospitals and primary care services, making data and information standardization problematic [8]. Robotics, sensors, and virtual reality have been explored for over a decade in many countries for their potential to augment human care-giving, but with little opportunity for uptake due to cost and complexity [9]. Telehealth has been shown to have a promising place in aged care, especially since the COVID-19 pandemic, but funding and service supply remain problematic [10]. Nevertheless, digital transformation of the aged care sector is underway [11,12].

Despite this, digital skills of any kind rarely appear in aged care job descriptions or training programs. Further, national workforce strategies related to digital health capability scarcely address the digital skill shortages in the aged care sector. On one hand, there is hope that the sector will find the “right” staff for digitally enabled aged care and find ways to assure them of technology’s quality and trustworthiness [13,14]. On the other hand, there is uneasiness that roles and responsibilities may become invisible, peripheral, or “care-adjacent” in the process of moving to technological efficiency and sustainability [15,16]. In short, the introduction of technology has still-unknown implications for the evolution of work in the aged care sector, with possible effects ranging from displacing workers to supporting productivity in current roles or to elevating responsibilities and recognition. However, much of the research to date into aged carers’ digital experiences and expectations has had a short-term focus on technology implementations rather than consequences and outcomes [17,18].

Working with technology in particular ways can support specific well-being factors, including positive emotions, self-awareness, mindfulness, empathy, and compassion—so-called positive computing [19]—and there are reports that this approach can benefit aged care organizations and their workers [20]. But there is no guarantee that this will happen without a deliberate workforce strategy. This raises questions about how to proceed, which shape the aims of further research: How can digital transformation of the aged care sector be optimized through greater investment in the digital knowledge, skills, attributes, and attitudes of its workforce? How can such investment in turn improve working conditions and job satisfaction in the aged care workforce?

The authors explored these questions during a workshop that we facilitated at the July 2023 MedInfo conference in Sydney, Australia. There we presented our view that workforce research and development is needed to achieve optimal outcomes for workers in order to achieve these for service users as the aged care sector moves toward digital maturity. The ensuing discussions with workshop delegates contributed to refining the viewpoint we outline here.

## What Knowledge and Skill Building Is Required for Digital Aged Care Work?

Digital aged care work is likely to deliver the greatest benefits when needs for fundamental, complementary digital knowledge and skills are met in both paid and unpaid workers. Table 1 summarizes and juxtaposes the capabilities that we propose.

**Table 1:** Digital capabilities needed in the aged care workforce.

For paid work	For unpaid work
Knowing about developments in the digital environment in which health and care operate	Knowing about the existence of digital systems and tools, and the ways they can support care users and workers
Knowing how to adopt digital tools to help with care coordination and monitoring	Knowing how to use tools to support care closer to home and enhance care provision
Knowing the importance for care quality of collecting and interpreting digital data collected during episodes of care	Knowing how to navigate a complex aged care system across multiple digital platforms
Ability with general digital proficiency and literacy, including information security	Ability with general digital proficiency and literacy
Ability to evaluate online information critically	Ability to access and interpret information from multiple online sources
Ability to consider usability and empathy when using digital tools with patients and clients	Ability to learn to engage with technology and be comfortable with technology
Ability to interpret biometric data and help the patient or client to understand it	Ability to be connected and share health data between care consumers and providers
Ability to judge when technology is not needed as well as when it is	Ability to access and use technology to overcome isolation

There are parallel aspects of capability, with different levels of sophistication, in the needs of paid and unpaid workers. Accordingly, paid workers must be cognizant of not only their own capabilities, but also the capabilities of the unpaid workers with whom they partner. Aside from the digital capabilities of direct consumers of aged care services, paid workers must consider and collaborate with the capabilities of volunteer carers among family, friends and community members. Thus, to build the digitally capable human resources that will support healthy and dignified aging for all, the sector certainly must increase levels of digital literacy in the paid workforce; digital literacy being defined as the ability to use technology to communicate, retrieve, and evaluate information to make decisions [21]. However, the sector also must increase levels of active engagement in digital citizenship among both paid and unpaid workers; in the concept of digital citizenship, people are increasingly expected to use a range of technologies to access services of all kinds and to participate on all levels in society [22].

Various approaches are possible to support digital knowledge building and skill building for the paid workforce. There seem to be gaps and requirements in terms of both formally accredited training programs and less formal continuing professional development, as well as for both workplace-based learning and workplace-independent learning. The diverse and differentiated methods needed to uplift the digital capabilities of the unpaid workforce are an even broader challenge. Strategy and culture will have to change in the aged care sector to create an environment that encourages and supports all those who do the work to embrace learning and development for positive uses of technology. Among service providers, technology suppliers, industry and community associations, and government agencies, it is not yet clear where roles and responsibilities should sit for planning, implementing, and evaluating the changes that are needed to create and support a digitally capable aged care workforce.

Although these themes are similar to those arising in the mainstream health care workforce in some respects, they also recognize that the aged care ecosystem is distinctive and heterogeneous in terms of the array of partnerships among service providers, service users, and community members [23]. It may be useful to build a more detailed understanding of the digital capability gaps and needs in this sector by overlaying two other system-level concepts onto the idea of the aged care ecosystem—care ecosystems and workforce ecosystems. A care ecosystem is defined as an approach to configuring paid and unpaid workers, ambient assistive technologies, and digital data sharing as work-arounds for intractable workforce shortages in care, particularly in situations that do not call for advanced or intensive clinical services [24,25]. A workforce ecosystem is characterized by employees and contractors working interdependently with automated processes to achieve the goals of an organization and their personal goals; it is considered a guiding concept for the challenges of human resources management as artificial intelligence changes industries and societies [26,27]. A whole-system approach that acknowledges these concepts could provide a coherent framework to plan, implement, and evaluate the range of complementary measures that are needed for digitally enabled aged care work.

## Conclusions and Next Steps

Digital technologies and digital data have the potential to improve many aspects of the experiences of the people who receive aged care and the people who provide it. The context in which we seek to develop the digital capabilities of the aged care workforce is challenging. There are strong sustainability pressures on service providers. Public policies, strategies, and sentiments favor innovative, distributed care. Social and technological services and solutions are not joined up. Workers across the sector are stretched thin, whether they are highly trained professionals (eg, registered nurses), minimally trained caregivers (eg, health and personal care assistants), or informal caregivers (eg families, friends, and neighbours).

Understanding how to build digital capability in this workforce is complex and multifaceted, with many unknowns. Inclusion is one: What learning and development methods are most efficient and effective to enable digital skills across the entirety of this workforce? Empowerment is another: How can aged care workers be enabled to advocate for and implement digital improvements that bring positive changes to care? Expertise is another: What career opportunities and leadership roles are appropriate to motivate digital aged care specialists and sustain their attention to workforce development?

Next steps for the work in this field include reviewing the literature to distill evidence from reports of small-scale efforts, workforce research, and development that engages real-world settings and stakeholders, as well as trials of scalable learning and professional and community development programs. To progress this research requires new collaborations among aged care workers, service provider organizations, aged care technology vendors, and digital health workforce researchers. We can improve the current standing of digital skills in the aged care workforce only if we first clarify what the workforce needs from education providers, employers, technology suppliers, and policy makers to enable and motivate them to use existing and emerging technologies safely and wisely. This work will provide important information to design learning and development that enhances digital capability, and it will establish expectations that allow us to evaluate changes in workforce status and performance as an integral part of the digital transformation of aged care.
